# Erector spinae plane block for perioperative pain management in neurosurgical lower-thoracic and lumbar spinal fusion: a single-centre prospective randomised controlled trial

**DOI:** 10.1186/s12871-023-02130-z

**Published:** 2023-05-30

**Authors:** Daniele Bellantonio, Giuliano Bolondi, Francesco Cultrera, Giorgio Lofrese, Lorenzo Mongardi, Luca Gobbi, Andrea Sica, Carlo Bergamini, Lorenzo Viola, Andrea Tognù, Luigino Tosatto, Emanuele Russo, Domenico Pietro Santonastaso, Vanni Agnoletti

**Affiliations:** 1 Anesthesia Unit - Ospedale Bufalini, viale Ghirotti 286, Cesena, FC 47521 Italy; 2 Neurosurgery Unit - Ospedale Bufalini, viale Ghirotti 286, Cesena, 47521 Italy; 3 Anesthesia Unit, Istituto Ortopedico Rizzoli, Via Nazionale Ponente 5, Argenta, FE 44011 Italy

**Keywords:** Early recovery after surgery, Erector spinae plane block, Neurosurgery, Postoperative pain, Regional anaesthesia, Spine, Spinal fusion

## Abstract

**Background:**

Erector spinae plane block is a locoregional anaesthetic technique widely used in several different surgeries due to its safety and efficacy. The aim of this study is to assess its utility in spinal degenerative and traumatic surgery in western countries and for patients of Caucasian ethnicity.

**Methods:**

Patients undergoing elective lower-thoracic and lumbar spinal fusion were randomised into two groups: the case group (n = 15) who received erector spinae plane block (ropivacaine 0.4% + dexamethasone 4 mg, 20 mL per side at the level of surgery) plus postoperative opioid analgesia, and the control group (n = 15) who received opioid-based analgesia.

**Results:**

The erector spinae plane block group showed significantly lower morphine consumption at 48 h postoperatively, lower need for intraoperative fentanyl (203.3 ± 121.7 micrograms vs. 322.0 ± 148.2 micrograms, p-value = 0.021), lower NRS score at 2, 6, 12, 24, and 36 h, and higher satisfaction rates of patients (8.4 ± 1.2 vs. 6.0 ± 1.05, p-value < 0.0001). No differences in the duration of the hospitalisation were observed. No erector spinae plane block-related complications were observed.

**Conclusions:**

Erector spinae plane block is a safe and efficient opioid-sparing technique for postoperative pain control after spinal fusion surgery. This study recommends its implementation in everyday practice and incorporation as a part of multimodal analgesia protocols.

**Trial registration:**

The study was approved by the local ethical committee of Romagna (CEROM) and registered on ClinicalTrials.gov (NCT04729049). It also adheres to the principles outlined in the Declaration of Helsinki and the CONSORT 2010 guidelines.

## Background

Erector spinae plane block (ESPB) is a recent locoregional anaesthetic technique [1]. Due to its safety and simplicity of execution, it has been extensively applied in several surgeries. The mechanism of action of ESPB remains imperfectly understood due to variable patterns of anaesthetic spread over the fascial layers. A well described and consistent finding, however, is the spread of the local anaesthetic along the dorsal rami, making this technique specifically appealing for spine surgery [2].

Despite the increasing interest in ESPB for spinal procedures, early publications were limited to case series or small trials [3–5], sometimes with ESPB used only as a postoperative rescue strategy [6]. The majority of more recent trials are from Asia: varied general anaesthesia (GA) techniques and drugs, together with a different socio-cultural approach to pain, may reduce the significance of these studies in Western Countries. These trials have focused exclusively on lumbar spine surgeries, involving three or fewer vertebral levels. The most frequent ESPB approach reported aims at targeting the transverse process of the L3 vertebra, independently from the lumbar spinal levels instrumented, relying on the spread of local anaesthetics [6–10]. The comparative advantages of different blocks, such as the thoracolumbar interfascial, is still animating scientific debate [11, 12]. In the context of multimodal analgesia, ESPB might play a crucial role in improving the postoperative experience of patients undergoing spinal fusion.

In Europe, the incidence of spinal arthrodesis is reported to be 20–33 per 100,000 person-years [13, 14], with a constant increase over the last 20 years. The average cost for these interventions and the following hospital stay is at least 8–10,000 € [15], while the cost of the whole diagnostic and therapeutic path can reach 15,000 € [16], with higher costs in north-European countries. Hospital stay usually ranges from 5 to 8 days depending on the invasiveness of the surgical approach [15]. The possibility to identify novel analgesic strategies may assume clinical and economic relevance when considering the sustainability of these procedures.

The hypothesis of this study is that ESPB might become a fundamental technique for reducing postoperative pain, perioperative opioid consumption, and the patients’ wellbeing after posterior lower-thoracic or lumbar spinal fusions, which are among the most painful surgeries to date [17]. The primary outcome evaluated was total opioid consumption at 48 h after surgery. The secondary outcomes assessed were pain, by the numeric pain rating scale (NRS) at rest, length-of-hospital stay (LOS) and satisfaction of the patients. NRS at 2, 6, 12, 24, and 48 h was assessed by trained nurses from the neurosurgical ward.

## Methods

### Study population

The current study was approved by the local ethical committee of Romagna (CEROM), registration number 1220 version 1.0, on 08/10/2020, and registered on ClinicalTrials.gov (NCT04729049) on 28/01/2021. It also adheres to the principles outlined in the Declaration of Helsinki. The enrolment phase was between February 2021 and June 2022. Our centre is a hub for neurosurgery and neuro-intensive care for a population of approximately 1,000,000, provided from a 32-bed neurosurgery unit, two neurosurgical theatres 5 days-per-week for elective surgeries (plus emergency activity), and approximately 500 spine surgeries per year.

The inclusion criteria were: (1) adult patients (between 18 and 85-year old) undergoing elective or urgent (within days from diagnosis) spinal fusions; (2) spinal fusions involving up to 4 consecutive vertebral spaces at thoracic or lumbar levels; (3) patients classified according to the American Society of Anesthesiology (ASA) as I, II, and III.

The exclusion criteria were: (1) allergy to local anaesthetics; (2) infection at the puncture site; (3) coagulopathy; (4) denial of consent from the patient; (5) need for emergent surgery (within hours from diagnosis); (6) patients classified as ASA IV or above; (7) chronic use of opioids; (8) patients who underwent previous spine surgery; (9) body mass index above 40.

Randomization was ensured by an informatically generated random sequence run by a clinician not involved in the study. Patients were informed during the preoperative anesthesiology evaluation, then they signed the informed consent forms at the preoperative re-evaluation the evening before surgery: 30 patients fulfilled the inclusion criteria during the study period and were enrolled, providing 15 cases and 15 controls. The ESPB group received ESPB plus intravenous analgesia, while the control group received intravenous analgesia only. Since ESPB was performed during GA, the control group did not receive any placebo to simulate ESPB.

### Clinical procedure

Preoperative visits were performed by skilled anaesthetists via the usual ambulatory path or the evening before for the urgent surgeries. Blood tests were performed following internal procedures: cell count, coagulation, renal and hepatic function were always included.

Before GA induction, an intravenous catheter was placed and electrocardiography, oscillometric blood pressure cuff and pulse oximetry were monitored. GA was induced with 2% propofol 2 mg kg^− 1^, fentanyl (1.5–2 mcg kg^− 1^) before induction, and rocuronium (5 mg kg^− 1^). After GA induction a second peripheral intravenous catheter was placed and invasive blood pressure monitored upon clinical indications. Propofol continuous infusion based on bispectral index guidance (target range, 40–60) and rocuronium based on train-of-four or surgeon requirements were used for maintenance of anaesthesia. Fentanyl (1 mcg kg^− 1^) was repeated when heart rate or mean arterial blood pressure increased by 20% with respect to the basal state of the patient.

**Fig. 1:**
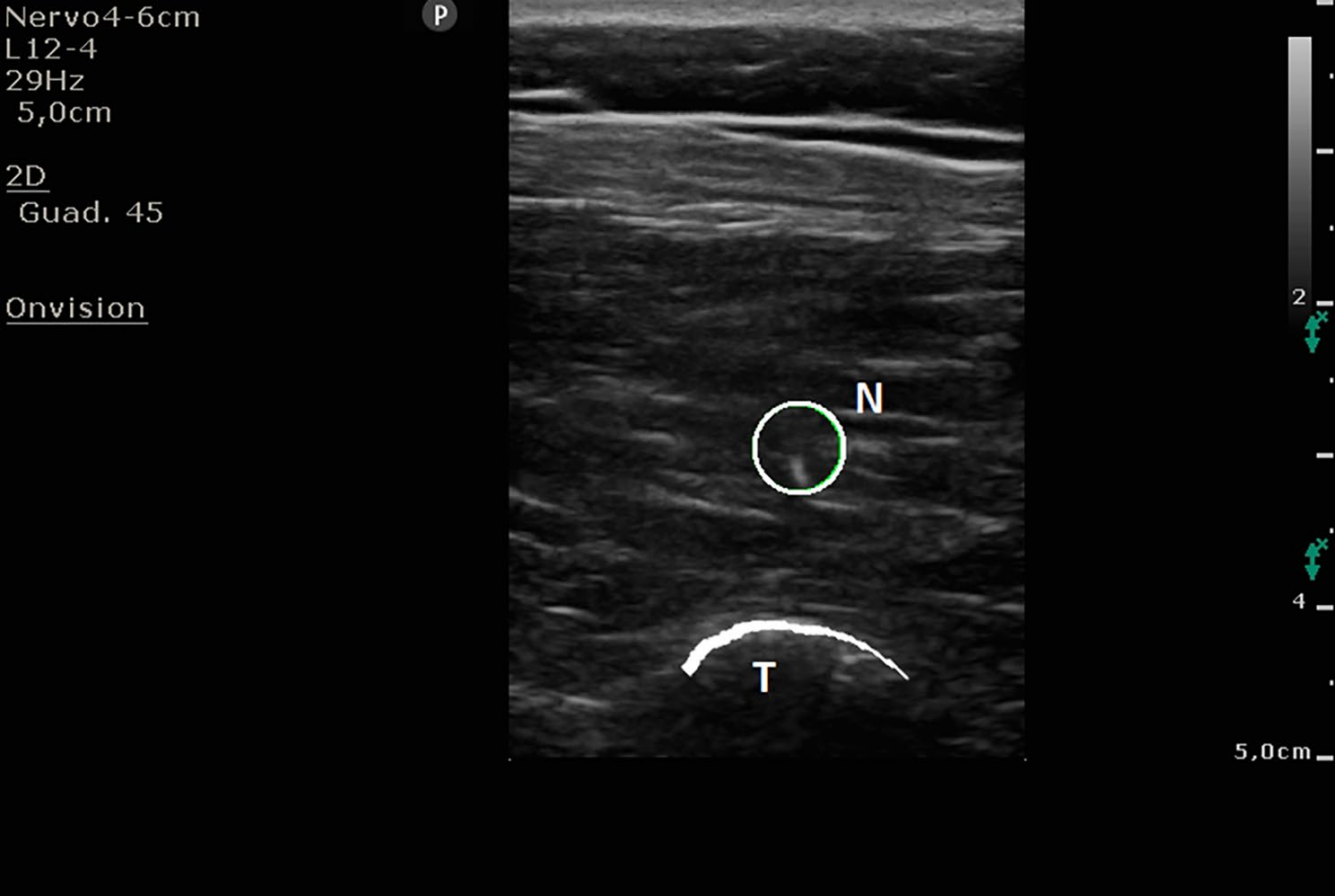
out-of-plane execution of ESPB N: needle tip in out-of-plane view; T: transverse process

**Fig. 2:**
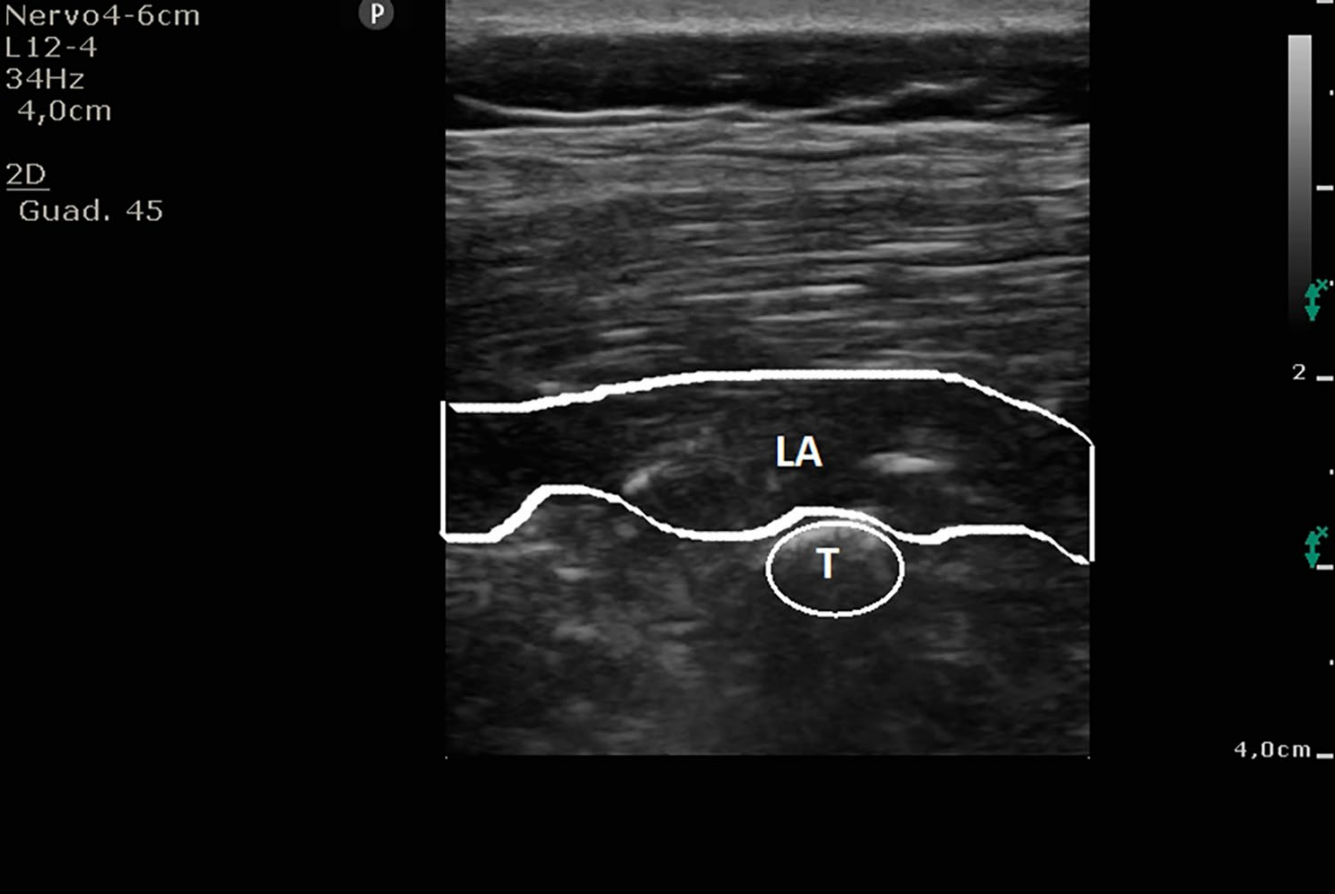
anaesthetic spread visualisation LA: local anaesthetic spread below the erector spinae; T: transverse process

Due to logistic reasons, ESPB was performed after GA induction, with the patient in prone position, and exploiting the intraoperative fluoroscopy to identify the exact levels to include in the spinal fusion. At this point, in all cases, the same five trained anaesthetists performed the ESPB and the same three surgeons conducted the intervention. Ultrasound guidance (SonoSite M-Turbo, SonoSite Inc., Bothell, WA; Philips CX50, Netherlands) with a 15 − 6 MHz linear probe or a 5 − 2 MHz convex probe depending on the depth of the transverse process, and a 22 G x 80 mm needle (Echoplex +, Vygon, Ecouen-France) were used for the ESPB. The transducer was positioned in a longitudinal orientation to obtain a parasagittal view. ESPBs were performed with sterile technique and an out-of-plane approach, targeting the median transverse process of the vertebral levels instrumented (Fig. 1). When the needle came in contact with the transverse process, ropivacaine 0.4% (160 mg) and dexamethasone 4 mg in a 20 mL of 0.9% sodium chloride (NaCl) volume were injected on each side. Anaesthetic spread was observed with sonographic insonation (Fig. 2). This procedure was always performed during the surgical scrub and the sterile preparation of surgical instruments, avoiding prolongation of the surgical time and systematically allowing 15–20 min to pass between the block and the surgical incision.

Postoperative analgesia was the same for all patients: a starter bolus with morphine (4 mg) and acetaminophen (1 g) 30 min before the surgical end of the intervention, then they were connected to a patient-controlled analgesia (PCA) pump (CADD-Solis, ICU Medical Inc., San Clemente, CA, USA) filled with 50 mg of morphine and allowing 1 mg boluses at a maximum of 15 min intervals with no background infusion. GA was concluded by interrupting propofol infusion after the suture was concluded, allowing the patients to recover just after medications and supination. After extubation, the patients were kept under observation until they reached an Aldrete score ≥ 9 and then returned to the neurosurgical ward. and then returned to the neurosurgical ward. The functioning of the PCA and the NRS scale use were explained to the patients the day before intervention and repeated after awakening from GA. Paracetamol (1 g) was set every 8 h and rescue IV ketoprofen (100 mg) was allowed.

### Clinical assessment

Primary and secondary outcomes have been stated at the end of the background section.

Patients,, nurses assessing the NRS, and the statistician were blinded with regard to the anaesthetic technique used. The anaesthetists performing the block were not blinded: they were caring for the patients during the intervention and collected the intraoperative data but were not involved in postoperative PCA data collection and statistical analysis. The surgeons were not blinded but also not involved in postoperative evaluations and statistical analysis.

There were some limitations in the clinical informatic system such as the inability to retrieve records about postoperative nausea and vomiting (PONV), the use of ketoprofen rescue doses, and the number of attempted doses of morphine with the PCA.

### Statistical analysis

Statistical analyses were performed using the software IBM SPSS (version 22.0, Armonk, New York, USA). Sample size calculation was based on a few internal pilot cases independently performed before the beginning of this protocol; the primary endpoint was a continuous variable (postoperative morphine consumption), alfa was set 0.05 and power 0.9, resulting in a total of 60 procedures to be performed. Data are reported as mean with standard deviation (std. dev.), median with interquartile range (IQR), or number and percentage depending on the underlying distribution. After normality distribution (Shapiro-Wilk) and homoscedasticity (Bartlett’s) tests, Mann-Whitney tests were used for statistical analyses of the samples which were non-parametric. Results were considered to be statistically significant if p ≤ 0.05.

## Results

Fifteen ESPB and 15 control procedures were performed between February 2021 and June 2022. The estimated sample size indicated on ClinicalTrial.gov was double (60) the enrolled sample size (30), but we had to conclude the study early, because of the delays with respect to the schedule mostly due to the COVID-19 pandemic period (the study was expected to end in December 2021).

**Fig. 3:**
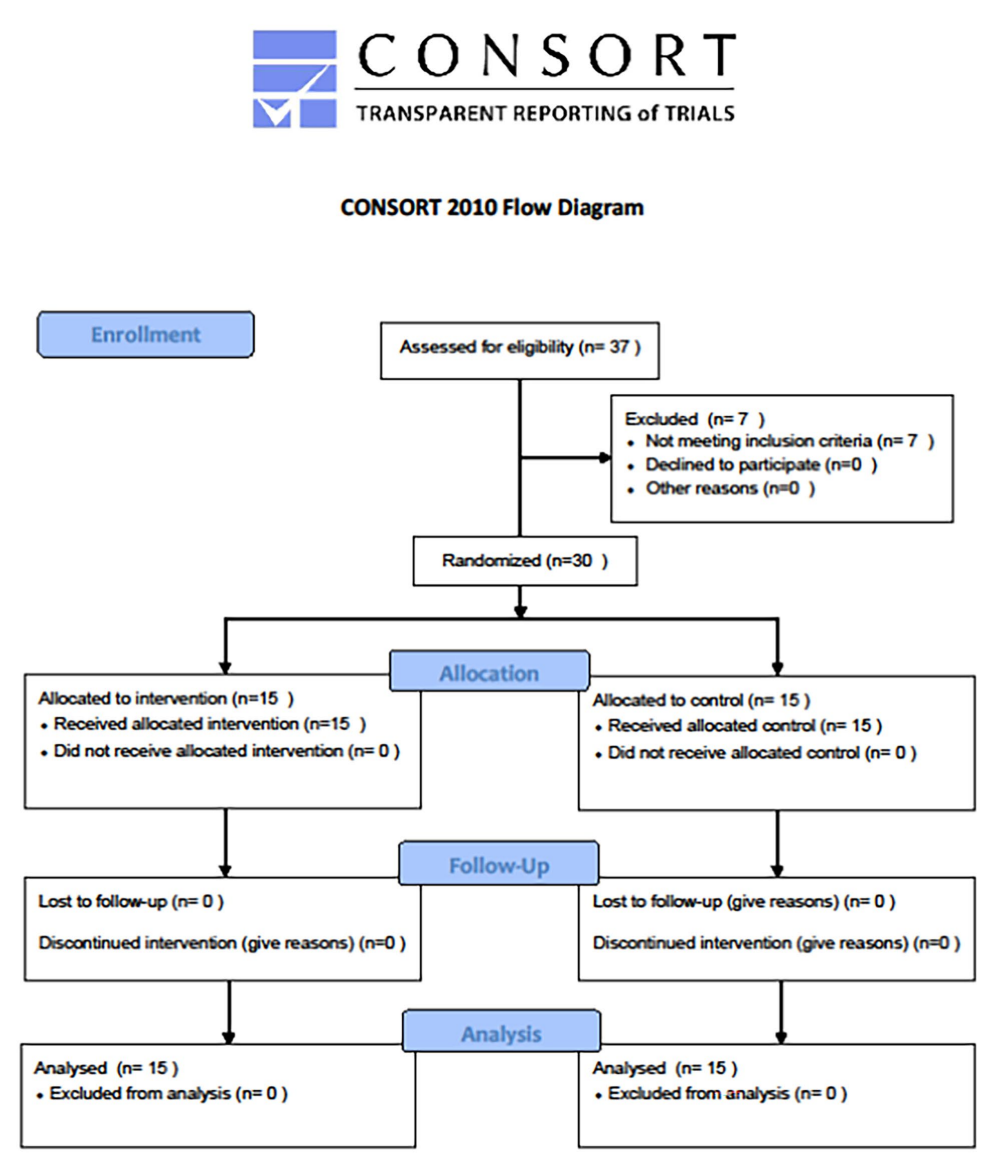
CONSORT flowchart of the study

The study adheres to the CONSORT guidelines for randomised controlled clinical trials, the CONSORT flowchart is reported in Fig. 3. The descriptive analysis between the two study groups is shown in Table 1. No significant differences were observed in terms of age, gender, body mass index, ASA score, and the number of spinal levels fused. Among the patients, 29 were Caucasians, while only one was North-African (randomised to the morphine-only group). The spinal arthrodesis ranged from T11 to S1 in the ESPB group, with two patients instrumented at the thoracolumbar junction) and T10–S1 in the control group, with five patients were instrumented at the thoracolumbar junction). Table 2 summarises the primary and secondary outcomes of the study.

**Table 1:** Descriptive analysis of the ESPB group vs. control group (morphine-only postoperative analgesia)

		ESPB	Control	p-value
Patients	N (%)	15 (50)	15 (50)	1
Age (years)	Mean (SD)	54.6 (16.8)	60.4 (11.4)	0.367
Gender	Male N (%)	7 (46.7)	8 (53.3)	0.715
	Female N (%)	8 (53.3)	7 (46.7)	
BMI (kg·m^− 2^)	Mean (SD)	26.4 (5.7)	27.5 (3.7)	0.305
ASA	I - N (%)	2 (13.3)	1 (6.7)	0.496
	II - N (%)	9 (60.0)	7 (46.7)	
	III – N (%)	4 (26.7)	7 (46.7)	
Number of vertebral spaces involved in fixation	1 - N (%)	5 (33%)	4 (27%)	1
	2 - N (%)	6 (40%)	6 (40%)	
	3 or more - N (%)	4 (27%)	5 (33%)	

ASA, American Society of Anesthesiology score; BMI, body mass index; N, absolute number; SD, standard deviation;. p-value refers to the mean ± SD and is considered significant when < 0.05.

**Table 2:** Statistical analysis of the primary and secondary outcomes of the study

		ESPB	Control	p-value	missing
PCA total consumption: primary outcome (mg)	Median (25–75 IQR)	8.5 (5.5)	20 (14)	< 0.0001	-
Intraoperative fentanyl use (mcg)				0.021	-
	Median (IQR)	200 (150)	250 (295)		-
Intraoperative blood loss (mL)				0.870	-
	Median (IQR)	250 (100)	250 (337.5)		-
NRS at 2 h				< 0.001	-
	Median (IQR)	0 (1.75)	5 (2.75)		-
NRS at 6 h				< 0.001	-
	Median (IQR)	2 (1.75)	5 (2.75)		-
NRS at 12 h				0.002	-
	Median (IQR)	3 (1)	5 (1.75)		-
NRS at 24 h				0.004	-
	Median (IQR)	3 (2)	5 (2)		-
NRS at 36 h				0.002	-
	Median (IQR)	2 (1.75)	4 (1.75)		-
NRS at 48 h				0.527	19
	Median (IQR)	2 (1.5)	3 (1.63)		19
PCA 0–2 h (mg)				0.007	-
	Median (IQR)	0 (0)	2 (3)		-
PCA 2–6 h (mg)				< 0.0001	-
	Median (IQR)	1 (1.75)	3 (1.75)		-
PCA 6–12 h (mg)				0.002	-
	Median (IQR)	2 (1)	3 (1.75)		-
PCA 12–24 h (mg)				0.002	-
	Median (IQR)	2 (2.75)	5 (3.5)		-
PCA 24–36 h (mg)				0.005	-
	Median (IQR)	3 (2.75)	5 (4.75)		-
PCA 36–48 h (mg)				0.046	13
	Median (IQR)	4.5 (3.30)	3.5 (4)		13
LOS (days)	Median (IQR)	5 (4)	7 (7)	0.226	-
Patients’ satisfaction				< 0.0001	-
	Median (IQR)	8 (2.75)	6 (1)		-

LOS, length of hospital stay; mcg, micrograms; N, absolute number; NRS, pain numeric rating scale; PCA, patient controlled analgesia (mg of morphine used at any time-interval); SD, standard deviation; IQR, interquartile range. Patients’ satisfaction is expressed with a vote from 1 (extremely bad) to 10 (extremely good) by the patient on the day of PCA pump removal. p-value refers to Mann-Whitney analysis and is considered significant when < 0.05.

**Fig. 4:**
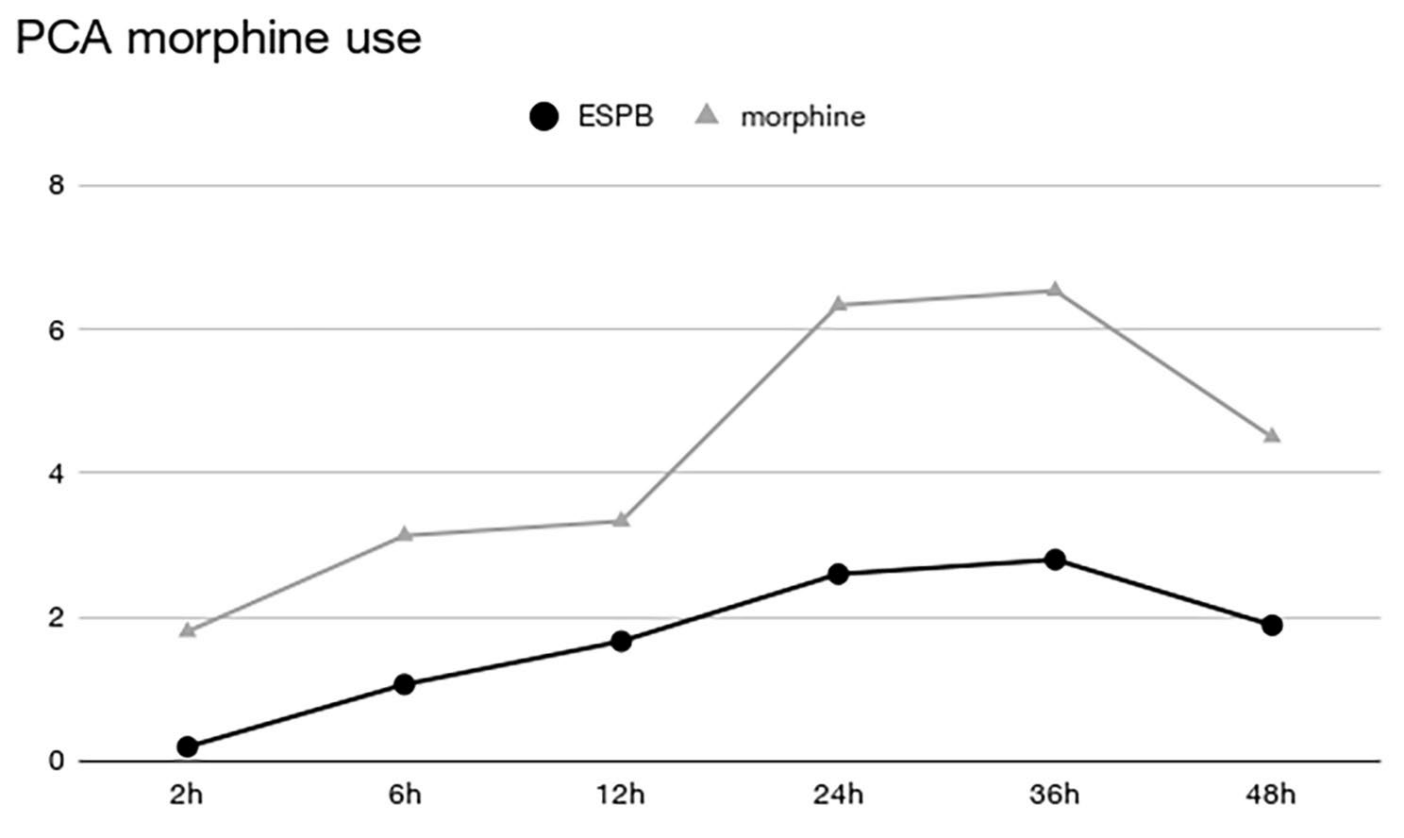
PCA morphine consumption x-axis, time; y-axis, milligrams of morphine; black line with dots, ESPB group; gray line with triangles, morphine-only group

The primary outcome was postoperative PCA morphine consumption: it was significantly lower at any time point during the first 48 h than in the control group. Fig. 4 demonstrates how this difference is consistent and relevant during the entire 48 h postoperative period.

Intraoperative fentanyl use was significantly lower in the ESPB group than in the control group (203.3 ± 121.7 mcg vs. 322.0 ± 148.2 mcg, p-value = 0.021). No differences in terms of intraoperative bleeding were detected, and no cases of hypotension or bradycardia were associated with the ESPB, testifying to the safety of the procedure. No significant side effects of ESPB were observed.

**Fig. 5:**
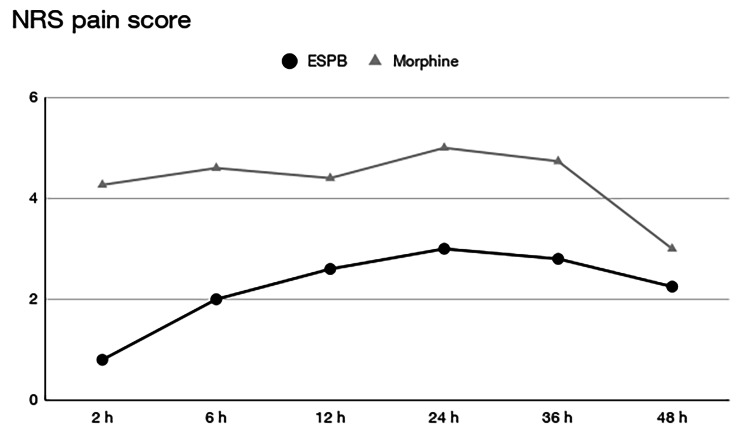
NRS pain score x-axis, time; y-axis, NRS; black line with dots, ESPB group; gray line with triangles, morphine-only group

NRS values were significantly lower in the ESPB group than in the control group up to 36 h after surgery. Fig. 5 demonstrates how these differences are more relevant during the first 12 h after surgery.

Despite not reaching statistical significance, LOS resulted 2 days longer in the control group, likely impacting the economic burden of the perioperative management of these patients. (7.67 ± 6.62 days vs. 9.67 ± 7.41 days, p-value = 0.192).

The final satisfaction expressed by the patients with a vote from 1 (awfully bad) to 10 (exceptionally positive) was significantly higher in the ESPB group than in patients managed with the standard analgesic protocol (8.4 ± 1.2 vs. 6.0 ± 1.2, p-value < 0.001).

## Discussion

### Relevant clinical outcomes

Our results demonstrate the benefits of performing ESPB before spinal fusion surgery in terms of perioperative opioid consumption and pain control and improving the hospital experience of patients. These benefits come at no additional cost in terms of clinical safety or significant side effects. ESPB can be safely and rapidly performed, without causing any delay in the surgical schedule.

Spinal arthrodesis is one of the most painful surgeries among the procedures commonly performed. The requirement for this operation is increasing in recent years, with significant costs in terms of quality of life and economic impact on the health system. This study demonstrates how ESPB can reduce the pain and opioid consumption of patients, at the same time reducing the stress and discomfort of the hospital experience. The reduction of opioid consumption also potentially reduces the risk of adverse effects, such as PONV, constipation, delayed awakening and mobilisation and the risk of chronic abuse, although specific data on these aspects were not available for statistical analysis.

Covering a wide variety of surgical interventions, including thoracic levels and traumatic patients that have never been addressed in previous studies, our protocol is strongly suggestive that ESPB should be implemented in clinical practice not just for degenerative lumbar spinal diseases, but also for traumatic fractures. To date, limited scientific literature is available on this topic describing such a broad approach: our results suggest the need for further specific clinical trials.

LOS did not differ significantly between the groups, but we cannot consider it a reliable indicator of ESPB efficacy because of the lack of an implemented early recovery after surgery (ERAS) protocol for fast-track surgery in our hospital and due to the many different types of surgeries included in this study. LOS in our study is in agreement with that reported in the scientific literature [15]. Rethinking a more comprehensive ERAS approach to spinal surgeries, including the ESPB, might reduce the economic burden on health systems, but new studies are needed in this direction.

Reduced pain and opioid consumption, together with a comprehensive ERAS approach and reduced LOS, should theoretically hasten the mobilisation of the patients and reduce their need for thromboprophylaxis or risk for venous thromboembolism, which is a crucial issue in neurosurgery [18, 19].

When this study was being planned, it was the only prospective randomised controlled study applying ESPB in spinal fusion surgery in western Countries. The only European study at that time was a retrospective registry analysis in the Netherlands [20] on patients undergoing posterior lumbar arthrodesis. That study only detected a slight reduction of NRS with ESPB and no differences in terms of opioid consumption and LOS, despite describing a clinical protocol similar to that of our study (ropivacaine 0.375%, in prone position after GA induction).

A recent study by Avis et al. [21] performed in France investigated a slightly larger sample size (50 patients), concluding that ESPB has no efficacy in terms of NRS and morphine consumption despite a similar locoregional protocol (ropivacaine 0.375% 20 mL per side, performed after GA induction). GA was maintained with ketamine and sufentanil and the total dose did not differ between the two study groups. This opposes our findings in the unblinded part of the study, that demonstrated improvements in the intraoperative analgesia due to ESPB. The analgesic effects of ketamine and a more complete multimodal analgesia (as supported by ERAS protocols) approach were adopted in the Avis study, probably diluting the effect of ESPB, which was just a single component of a comprehensive approach. They included lumbar spinal fusions of 2 or more levels exclusively, excluding spinal arthrodesis at thoracic levels; our inclusion criteria were wider and focused on the most common multi-level spinal surgeries involving traumatic fractures of the thoracolumbar spine. Interestingly, Avis et al. [21] assessed the three months postoperative pain and no long-term benefits from ESPB were detected.

Studies reporting ten other clinical trials developed in Asia were available and retrieved. The cultural approach and clinical susceptibility to pain of the different ethnicities involved, together with the differences in healthcare systems could limit their significance in Western Countries. The extreme variability of GA methods, ESPB techniques, and postoperative analgesia protocols and drugs used might limit their reproducibility and increase the number of possible confounders. Most of these studies did not include thoracic levels nor ASA III patients, focusing on different types of lumbar surgeries. Only the studies by Singh et al. [8], Zhang et al. [9], and Yayik et al. [10] included ASA III patients, while only three studies [7, 10, 20] used ropivacaine as a local anaesthetic for ESPB with a concentration similar to our study (0.3–0.4%, 20–25 mL per side), but without the addition of dexamethasone (4 mg per side).

Overall, these studies revealed similar results, with significant benefits in terms of pain scores and opioid consumption during the first 12–24 h following ESPB, but these beneficial effects faded in a shorter time than in our study. Only a few authors assessed the intraoperative use of opioids [12] showing lower pain scores in the ESPB group, and detected benefits in terms of decreased incidence of PONV, [12, 22, 23] need of rescue doses, [23] decreased incidence of LOS, [22] and reduced blood loss. [24] None has evaluated the satisfaction of the patients at the end of the 48 h-postoperative period.

As reported by the analysis of De Cassai et al., [25] the safety profile of the ESPB is extremely encouraging also at the thoracic level. The present study has not detected an increased rate of adverse effects. Further studies on ESPB and its implementation in ERAS protocols for spine surgery in degenerative and traumatic disease at thoraco-lumbar levels should not be delayed by unjustified concerns.

### Potential limitations and biases

The major limitations of our study are mostly due to the loss of clinical information. It was not possible to collect data about the exact duration of ESPB performance, the duration of GA, and surgical intervention from the operation theatre;. From the ward it was not possible to collect or retrieve data about PONV, rescue ketoprofen doses, required PCA doses (not just dispensed) and NRS on movement; considerable data were missing at 48 h, limiting their relevance. This was probably due to the non-academic nature of our institution, lacking dedicated research facilities and personnel, but this did not affect the measurement and reliability of primary and secondary outcomes. Anyway, this could also be interpreted as a strength of this study: the positive outcomes recorded were measurable and significant already during the everyday clinical practice, with no extra-fundings required, and presumably impact the real postoperative process of the patients.

The limited sample size was a consequence of the COVID-19 pandemic and the slowing down of daily clinical practice:. despite a 6-month extension of the study insurance, it was not possible to respect the expected enrolment pace.

Another possible source of bias was regarding the intraoperative use of fentanyl: the unblinding of the anesthesiologists to the ESPB procedure, might affect their propensity to administer the drug. All the other steps have been standardised and are reproducible by other authors. Finally, due to the monocentric nature of the study, the reproducibility might be reduced and the results affected by local practices.

## Conclusion

The ESPB proved to be a safe and effective approach in reducing postoperative opioid consumption up to 48 h after surgery, providing adequate control of postoperative pain, and significantly increasing the satisfaction of patients. Our study confirms the efficacy of ESPB in spinal fusions of thoracic and lumbar spinal levels, expanding the validity of these findings to Caucasian patients and to the healthcare systems of the Western world.

The extensive use of ESPB in thoracic and lumbar spine surgery is thus recommended.

Moreover, considering its limited cost, safety profile and lack of impact on the surgical theatre schedule, novel investigations and clinical trials expanding the application of ESPB to a wider range of thoracic and lumbar spinal surgeries, independently of their degenerative or traumatic etiologies, should be considered.

## Data Availability

The datasets generated and/or analysed during the current study are not publicly available to ensure higher levels of data safety and protection, but are available from the corresponding author on reasonable request.

## Abbreviations

ASA: American society of anesthesiology
ERAS: early recovery after surgery
ESPB: erector spinae plane block
GA: general anaesthesia
IQR: interquartile range
LOS: length-of-hospital stay
NRS: numeric pain rating scale
PCA: patient-controlled anaesthesia
PONV: postoperative nausea and vomit
